# Thermal Processing Map and Microstructure Evolution of Inconel 625 Alloy Sheet Based on Plane Strain Compression Deformation

**DOI:** 10.3390/ma14175059

**Published:** 2021-09-03

**Authors:** Yuelin Song, Jiangkun Fan, Xudong Liu, Peizhe Zhang, Jinshan Li

**Affiliations:** 1 State Key Laboratory of Solidification Processing, Northwestern Polytechnical University, Xi’an 710072, China; 2020200843@mail.nwpu.edu.cn (Y.S.); liu_xudong@mail.nwpu.edu.cn (X.L.); zhangpeizhexgd@163.com (P.Z.); ljsh@nwpu.edu.cn (J.L.); 2 Innovation Center, NPU·Chongqing, Chongqing 401135, China; 3 National & Local Joint Engineering Research Center for Precision Thermoforming Technology of Advanced Metal Materials, Xi’an 710072, China

**Keywords:** Inconel 625, constitutive equation, processing map, recrystallization, microstructure evolution

## Abstract

Plane strain compression tests were used to study the deformation behavior of an Inconel 625 alloy sheet at various temperatures and strain rates. The peak stress was selected to establish the constitutive equation, and the processing maps under different strains were drawn. The results show that the effective stress–strain curve of Inconel 625 has typical dynamic recrystallization (DRX) characteristics. With the increasing deformation temperature and the decreasing strain rate, the softening effect is significantly enhanced. The parameters of the constitutive equation are calculated, and the average error of the constitutive equation is 5.68%. Through the analysis of the processing map, a deformation temperature of 950–960 °C with a strain rate of 0.007–0.05 s^−1^ were determined as the unstable region, and obvious local plastic-rheological zones were found in the unstable region. The optimum deformation condition was found to be 1020–1060 °C/0.005–0.03 s^−1^. Through electron backscattered diffraction (EBSD) characterization, it was found that both the increase of temperature and the decrease of strain rate significantly promote the recrystallization process. At a low strain rate, the main recrystallization mechanism is discontinuous dynamic recrystallization (DDRX). It is expected that the above results can provide references for the optimization of the rolling process and microstructure control of an Inconel 625 alloy sheet.

## 1. Introduction

Due to the stable matrix elements and the large extent of the γ″ phase, Inconel 625 has excellent high-temperature strength and structural stability [1,2,3,4]. In addition, Inconel 625 has good machinability and weldability [5,6]. Therefore, Inconel 625 can be easily processed into various components such as plates, bars, pipes, wires, and strips [7]. However, due to its complex composition, strong deformation resistance, and narrow range of hot-forming parameters, it is easy to segregate during preparation, and there are some problems in rolling processes, such as uneven distribution of stress or temperature, and fracture [8].

In order to solve various problems in processing, some scholars have studied the deformation behavior of Ni-based superalloys recently [9,10,11,12]. Chen et al. [13] studied the hot-deformation behavior of GH4169 under different strain modes. They found that when the strain rate remains high in the first stage and then becomes low in the second stage, the true stress decreases with the sudden decrease of the strain rate. Compared with the equivalent constant strain rate, there is no difference in the final stress. Both constant-strain-rates and changed-strain-rates GH4169 have the same recrystallization mechanism, while the recrystallization grain size and volume fraction of the former is between those of the latter. Jia and Tao et al. have carried out uniaxial compression and high-speed compression experiments on cylindrical Inconel 625 respectively [14,15]. Under different temperatures and strain rates, the real stress–strain curve shows an obvious steady-state flow rule, and the adiabatic heating effect decreased with decreasing temperature and increasing strain rate. Moreover, many new models have been developed to describe the high-temperature rheological behavior of nickel base alloys, such as the improved Arrhenius model [16], constitutive model based on physical mechanism [17], artistic neural network (ANN) model [18], deep belief network (DBN) model [19], multigene genetic programming (MGGP) [20], and so on. All of those have good prediction effects with different accuracy.

Some scholars have also studied the microstructure evolution and recrystallization behavior of the alloy [21,22,23]. By combining T-EBSD with transmission electron microscopy (TEM), Sun Fei [24] accurately studied the microstructure evolution of recrystallization process of U720Li disc superalloy. They found that with the increasing rate of strain, the slip dislocations tended to gather into the wall and form subgrain boundaries. Then, the dislocation wall was connected with the local primary γ′ and combined to form subgrains. Kumar et al. [25] found that as for the advanced P/M Ni-based superalloy, the DRX reached the maximum at a higher deformation temperature. While at a lower deformation temperature, the DRX was limited and the structure highly deformed. Sun et al. [26] developed the effect of microstructure on recrystallization evolution of Inconel 625. Through high-resolution EBSD and transmission electron microscopy, they found that the δ phase can promote the progress of CDRX. In addition, Lamellar carbides (NbC) have a pinning effect on dislocations, which limits the growth of DRX grains in DRX process. Jiang et al. [27] simulated the microstructure evolution of the 690 alloy pipe-forming process by using the finite element method (FEM). The results show that the established numerical model can accurately simulate the dynamic recrystallization, subdynamic recrystallization, and grain-growth behavior of the 690 alloy pipe-forming process. Sun et al. [28] developed a model for predicting the microstructure evolution of nickel base alloy during hot deformation by 2D cellular automaton (CA). This method uses deterministic or stochastic element evolution rules, which are not limited in time and space and do not need to establish complex differential equations. The method can easily simulate the nucleation position, orientation, and growth process of grains, and provides great help for the study of recrystallization.

At present, the research on the hot deformation of nickel base superalloys mainly focuses on the uniaxial compression of cast cylindrical specimens, but there is little on the plane strain compression of rolled plates. Limited by the single deformation mode of the former, it is difficult to reflect the deformation characteristics of the alloy in multiple modes, and it is even more difficult to optimize the processing technology. With the increasing demand for plate and strip, the simulation of rolling behavior has become a part that cannot be ignored. However, the cylinder compression experiment cannot reflect the deformation behavior of the actual sheet. Due to the effect of friction, it is prone to uneven deformation and a “bulging” phenomenon. In addition, when the strain is large, it may lead easily to the abnormal increase of flow stress, which has an impact on the measurement of flow stress and the observation of microstructure [29]. In contrast, the geometric profile of the deformation zone during plane strain compression is closer to that of flat rolling. This method can well reflect the deformation state, heat conduction, or other information in the rolling process, and the measurement of rheological stress is more accurate [30]. In order to study the rolling deformation behavior and microstructure evolution of the Inconel 625 plate more accurately and systematically, the plane strain compression of a rolled rectangular plate at minimal strain rate was carried out, and some novel results were obtained. This complements the related work of observing a rolled Inconel 625 plate under different compression conditions and minimal strain-rate range. It is conducive to a more accurate and in-depth understanding of the rolling process of Inconel 625 sheets, which is of great significance for guiding production, reducing cost, and subsequent research.

## 2. Experimental

### 2.1. Plane Strain Isothermal Compression Test

An Inconel 625 industrial hot-rolled sheet was selected in this experiment, and its chemical composition is shown in Table 1.

**Figure 1:**
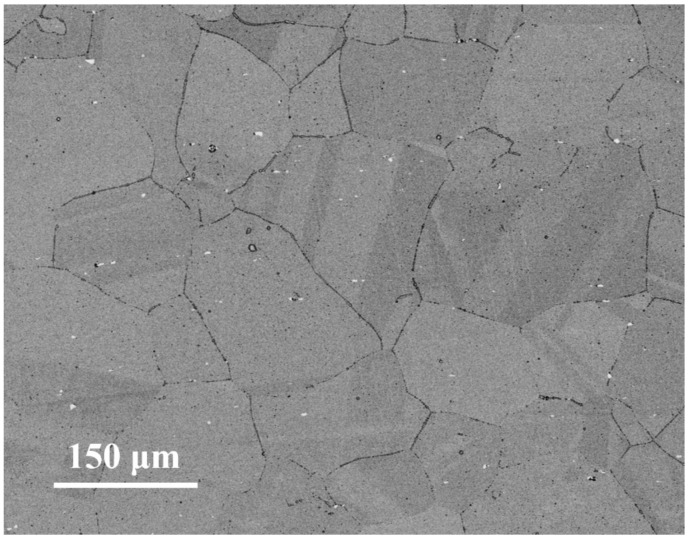
The original microstructure of annealed Inconel 625 characterized by BSE.

**Figure 2:**
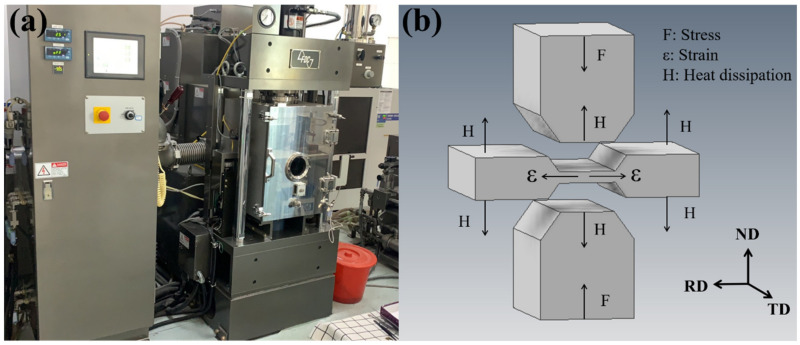
Experimental instruments and compression mode: (**a**) Thermecmastor-Z dynamic thermomechanical simulation testing machine, (**b**) schematic diagram of plane strain compression.

After annealing at 1150 °C for 50 min, the sheet was processed into a rectangular specimen with a size of 20 mm × 15 mm × 10 mm, and the original microstructure of the sample is shown in Figure 1. It can be seen that the annealed sample contains a large number of equiaxed and uneven grains, accompanied by the precipitation of white Nb-rich particles, and that twinning occurs in the grain. The plane strain compression test was carried out using a Thermecmastor-Z dynamic thermomechanical simulation testing machine (Fuji Electronics Industry Co., Ltd., Osaka, Japan). This equipment uses high-frequency heating and electric heating to heat the sample at the same time, and the thermal deformation is controlled by oil pressure. The data were collected by computer and program controller, and then the real stress–strain curve and processing map were drawn using the Origin 2021b software (OriginLab Corporation, Northampton, MA, USA). The hot-pressing experiments were carried out at 950–1100 °C and strain rates of 0.001–0.05 s^−1^, and the strain of all samples was 0.7. The compression equipment and compression mode of this experiment are shown in Figure 2, where RD, TD, and ND represent rolling direction, transverse direction, and normal direction. In the actual compression process, the width of the platen is narrow, and there is graphite gasket lubrication between the platen and the sample. Therefore, the compressive stress P and strain $\varepsilon_{y}$ in plane strain compression can be converted into effective stress σ and strain ε, according to Equations (1) and (2).

(1) $$\sigma =\frac{\sqrt{3}}{2}P$$

(2) $$\varepsilon =\frac{2\sqrt{3}}{3}\varepsilon_{y}$$

### 2.2. Microstructure Analysis

Firstly, the 80 # SiC sandpaper was used to remove the scratch and heat treatment oxide scale on the surface of the sample. Then Samples were ground up to 2000 # sandpaper and polished on an MP-1A metallographic polishing machine (Caikon Optical Instrument Co., Ltd., Shanghai, China), in which the disc was diameter 230 mm and the maximum power was 400 W. After ultrasonic cleaning, vibration polishing was carried out on a Buehler Vibromet2 polisher (Buehler, Lake Bluff, IL, USA) for 8 h. As Inconel 625 is a corrosion-resistant alloy, aqua regia was prepared as the corrosion solution, in which the ratio of hydrochloric acid and nitric acid was 3:1. After corrosion, the sample was cleaned with anhydrous ethanol and observed on an Olympus GX71 metallurgical microscope (Olympus Corporation, Tokyo, Japan).

The microstructure of the Inconel 625 was observed and analyzed by a ZEISS Gemini 500 field emission scanning electron microscope (Carl Zeiss Jena, Oberkochen, Germany), and the effect of thermal pressure deformation on tissue grain size was investigated by characterization with EBSD. Among the characteristics, the accelerating voltage was 20 kV and the step size was 0.7 μm. Finally, the results were analyzed using the Channel 5 5.0.9.0 software (Oxford Instruments, Oxford, UK).

## 3. Results and Discussion

### 3.1. The Effective Stress–Strain Curves and Flow Behavior

**Figure 3:**
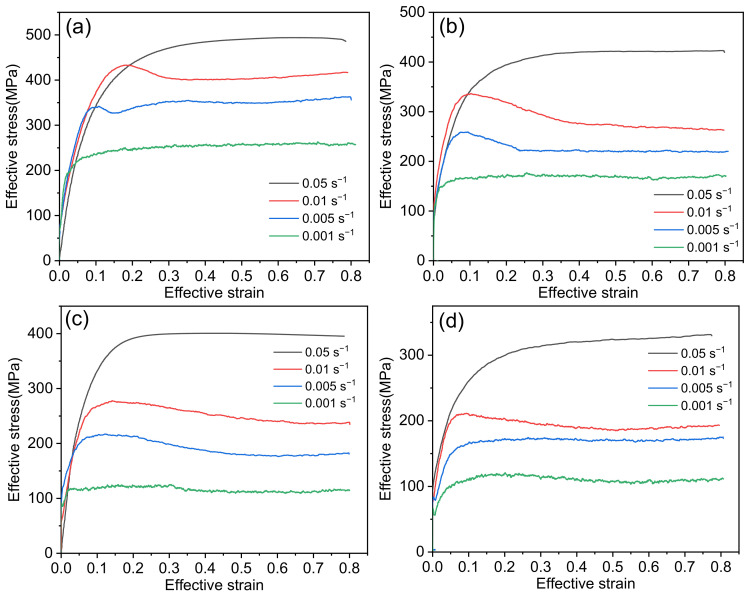
Stress–strain curves of Inconel 625 of 0.7 at (**a**) 950 °C, (**b**) 1000 °C, (**c**) 1050 °C, (**d**) 1100 °C.

Figure 3 shows the effective stress–strain curves of Inconel 625 under different hot-pressing parameters. In the initial stage of alloy deformation, the stress increases in a nearly straight line and reaches the peak value rapidly. This phenomenon indicates that an obvious work-hardening effect occurs during the deformation process. Significantly, at low strain rate, the curve appeared to have jagged fluctuations after reaching the peak value. Meanwhile at high strain rate, the curve still rises gently after yielding until it enters a stable flow stage. The main reason for this is that at low strain rate, dynamic strain aging occurs. That is, many interstitial atoms gather near the dislocation to form a Cottrell atmosphere, which plays the role of pinning the dislocation. When the dislocation starts to move, the stress decreases. This process repeats continuously, which results in serrated undulations in the stress–strain curve [12,31]. At the initial stage of deformation, the hardening effect plays a dominant role and the flow stress increases. When the stress reaches the peak value, the DRX of Inconel 625 is obviously strengthened, and dynamic softening begins to take the lead. The flow stress gradually decreases and then remains stable. At this time, the dynamic balance between work hardening and dynamic softening is maintained.

Additionally, the peak stress decreases with increasing strain rate. It is well-known that flow stress is essentially an interactive system of work hardening and recrystallization softening. At the same deformation temperature, with the increasing strain rate, the time needed to reach the same strain will be shorter. The recrystallized grains do not have enough time to nucleate and grow, and the DRX process is incomplete, thus the softening effect is relatively reduced. At the same time, the rate of dislocation formation is also increasing, and the high density of dislocations causes dislocation movement to be impeded. In comparison, the work-hardening effect is relatively enhanced, and the flow stress increases continuously. On the curve, it is shown as an increase in the peak stress.

In addition, it can be observed that with the increasing of deformation temperature, the peak stress decreases gradually. This is because, at the same strain rate, the diffusion rate of grain boundary increases with deformation temperature. Under the same deformation conditions, more dislocation will be activated, which promotes the recrystallization softening process. As a result, the peak stress is constantly reduced. Under the same strain rate, the degree of work hardening of the alloy is approximately the same, and the DRX behavior will soften the material [14]. Therefore, in a certain temperature range, a rise in temperature can promote the DRX process of Inconel 625.

### 3.2. The Establishment and Solution of Constitutive Model

It can be seen from the above that the flow stress is closely related to the deformation temperature and the strain rate $\dot{\varepsilon }$. Through the constitutive equation of materials, we can know the relationship of thermal deformation parameters in the process of thermal deformation, such as flow stress, deformation temperature, strain rate, and strain [32,33,34]. Therefore, the constitutive model can predict the rheological behavior of Inconel 625, and provide theoretical support for selecting suitable processing equipment and forming parameters. Combining with the modified Arrhenius equation of hyperbolic sine form, we usually use the Zener–Hollomon parameter (the temperature compensation deformation rate factor) proposed by Sellars and Tegart to describe the relationship between thermal deformation parameters.

(3) $$Z=\dot{\varepsilon }exp\left(Q/RT\right)=A_{1}\sigma^{n}\;\;\;\;\left(\alpha \sigma \leq 0.8\right)$$

(4) $$Z=\dot{\varepsilon }exp\left(Q/RT\right)=A_{2}exp\left(\beta \sigma \right)\;\;\left(\alpha \sigma \geq 1.2\right)$$

(5) $$Z=\dot{\varepsilon }exp\left(Q/RT\right)=A{\left[sinh\left(\alpha \sigma \right)\right]}^{n}\;(Full\;stress\;level)$$

(6) α=β/n

where ***σ*** represents the flow stress (MPa), $\dot{\varepsilon }$ represents the strain rate (s^−1^), ***R*** is the molar gas constant, 8.314 J/(mol*K), and *T* is the thermodynamic temperature (K). When *α*, *N*, *A*, and ***Q*** are known, the above equation can be used to describe the rheological properties of Inconel 625 during hot deformation. Through the study of thermal deformation data, the relationship between *σ* and *Z* can be described by exponential relation at a low stress level, such as Equation (3). Meanwhile, the relationship between *σ* and *Z* can be described by power exponential relation at a high stress level, such as Equation (4). In the actual production, the hot deformation of Inconel 625 is under the full stress level, and the relationship between ***σ*** and *Z* is shown in Equation (5).

**Figure 4:**
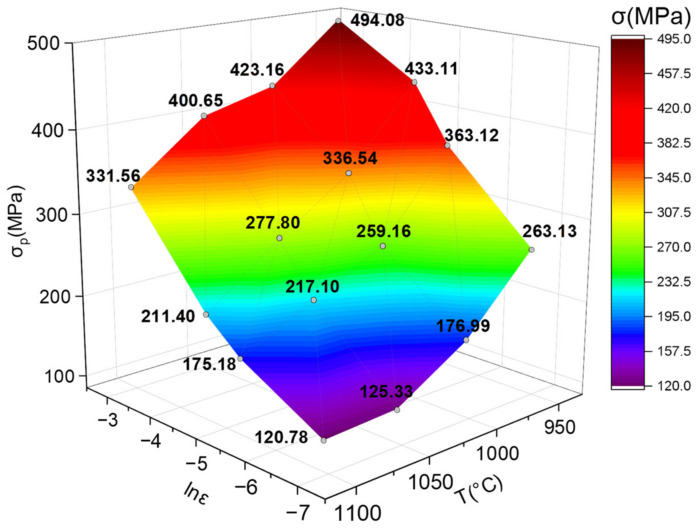
The peak stress at different deformation temperature and strain rate.

McQueen et al. [35] pointed out that for the material whose softening behavior is mainly DRX, the peak stress (*σ_p_*) or steady-state stress (*σ_s_*) is usually selected as ***σ*** for calculation and analysis. From the above analysis, Inconel 625 shows obvious DRX characteristics during hot deformation, so *σ* in the constitutive equation is marked *σ_p_*. The peak stress under different deformation temperature and strain rate is shown in Figure 4.

According to the flow stress model of the material during plastic deformation at high temperature, the correlation constants *n*_1_, *β*, *α* can be solved. Take the logarithm for both sides of Equations (3) and (4):

(7) $$ln\dot{\varepsilon }=lnA_{1}-\frac{Q}{RT}+n_{1}ln\sigma$$

(8) $$ln\dot{\varepsilon }=lnA_{2}-\frac{Q}{RT}+\beta \sigma$$

**Figure 5:**
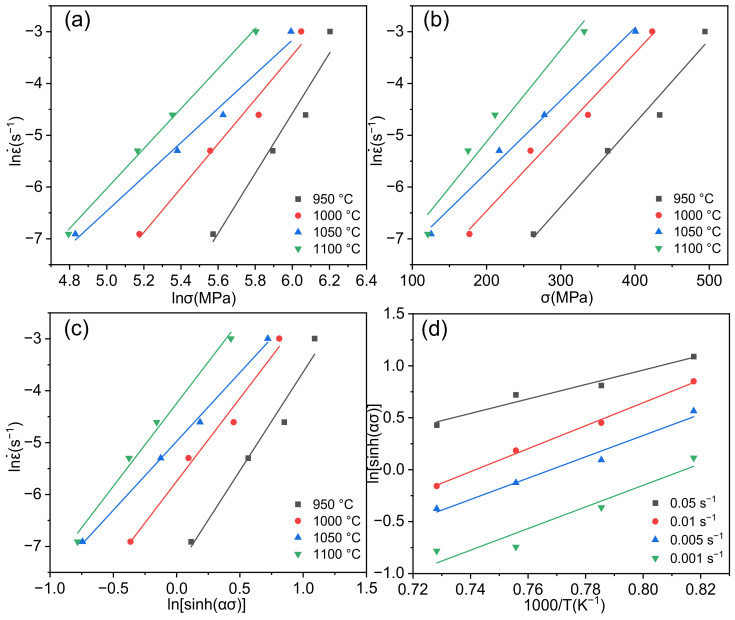
Relationship diagram of thermal deformation parameters. (**a**) $ln\dot{\varepsilon }$ and lnσ, (**b**) $ln\dot{\varepsilon }$ and σ, (**c**) $ln\dot{\varepsilon }$ and $ln\left[sinh\left(\alpha \sigma \right)\right]$, (**d**) $ln\left[sinh\left(\alpha \sigma \right)\right]$ and 1000/*T*.

It can be seen from the above equation that there is an obvious linear relationship between $ln\dot{\varepsilon }$ and *n*_1_, *β*. Plotting with $ln\dot{\varepsilon }$-*lnσ* and $ln\dot{\varepsilon }$-*σ* as the coordinate axis, we can get Figure 5a,b.

Then the slope is obtained by linear regression with the least square method. Calculating and taking the average, we find that *β* = 0.016, *n*_1_ = 4.31, Then we can get that *α* = *β*/*n*_1_ = 0.016/4.31 = 0.0037.

For full stress level, the Equation (5) can also be expressed as:

(9) $$\dot{\varepsilon }=A{\left[sinh\left(\alpha \sigma \right)\right]}^{n}exp\left(-\frac{Q}{RT}\right)$$

After obtaining the logarithm on both sides of Equation (9), the partial differential can be expressed as:

(10) $$Q=R\{\frac{\partial ln\dot{\varepsilon }}{\partial ln\left[sinh\left(\alpha \sigma \right)\right]}{\}}_{T}\cdot \{\frac{\partial ln\left[sinh\left(\alpha \sigma \right)\right]}{\partial \left(\frac{1}{T}\right)}{\}}_{\dot{\varepsilon }}$$

It can be seen from Equation (10) that when the deformation temperature is fixed, $ln\dot{\varepsilon }$ and $ln\left[sinh\left(\alpha \sigma \right)\right]$ have a linear relationship, so let *n*_2_ be the slope of this relationship. When the strain rate is constant, $ln\left[sinh\left(\alpha \sigma \right)\right]$ has a linear relationship with *T^−1^*, and let *K* be the slope of the relationship. The above is represented by Figure 5c,d.

After calculating, *n*_2_ = 3.21, *K* = 9.67, and ***Q*** = ***R*** × *n*_2_ × *K* =8.314 × 3.21× 9.67 = 258.22 kJ/mol.

Take the logarithm on both sides of Equation (5) to get:

(11) $$lnZ=\dot{ln\varepsilon }+\frac{Q}{RT}$$

(12) $$lnZ=lnA+nln\left[sinh\left(\alpha \sigma \right)\right]$$

**Figure 6:**
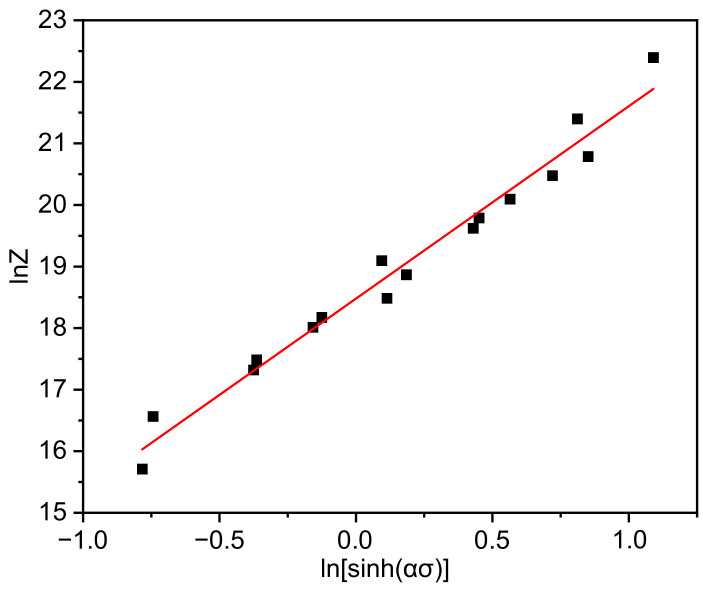
Relationship diagram of thermal deformation parameters lnZ and $ln\left[sinh\left(\alpha \sigma \right)\right]$.

From (12), we can see that there is a linear relationship between *lnZ* and *ln*[*sinh* (*ασ*)], and *lnA* is the intercept of this line. Calculate *lnZ* by Equation (11), and use the least square method and linear regression method to draw the corresponding linear relationship diagram as shown in Figure 6.

According to its intercept, we can get *lnA* = 18.48, *A* = 1.06 × 10^8^.

Bring all parameters into Equation (5), we can get the constitutive equation of Inconel 625 as follows:

(13) $$Z=\dot{\varepsilon }exp\left(2.58\times 10^{5}/RT\right)=1.06\times 10^{8}{\left[sinh\left(0.0037\sigma \right)\right]}^{3.21}$$

It can also be expressed by flow stress as:

(14) $$\sigma =272.97ln\left\{{\left(\frac{\dot{\varepsilon }exp\left(\frac{2.58\times 10^{5}}{8.314T}\right)}{1.06\times 10^{8}}\right)}^{0.31}+\sqrt{{\left(\frac{\dot{\varepsilon }exp\left(\frac{2.58\times 10^{5}}{8.314T}\right)}{1.06\times 10^{8}}\right)}^{0.62}+1}\right\}$$

**Figure 7:**
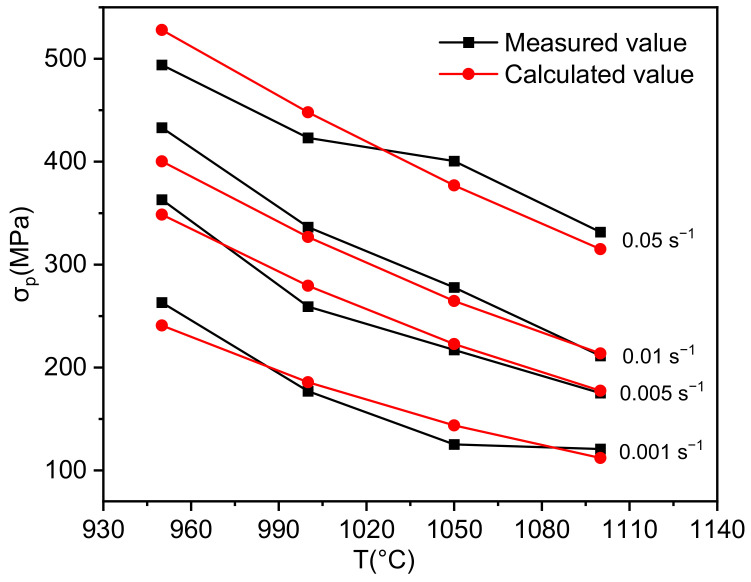
Comparison of measured and calculated peak stress of Inconel 625.

The peak stresses of Inconel 625 obtained under the experimental conditions are compared with the calculated value by using the constitutive equation, as shown in Figure 7. The average error is 5.68%, which verifies the applicability of the constitutive equation under this deformation condition.

It is worth noting that compared with other studies [36,37], the deformation activation energy of this experiment is low. Deformation activation energy is usually considered to reflect the difficulty of plastic deformation, and there are many influencing factors, such as microstructure (initial grain size, dislocation pinning effect [38], and the concurrent dynamic precipitation [35]) and deformation conditions (temperature [39], strain rate [40], and accumulated deformation [41]). On the one hand, the deformation mode and stress state of plane strain compression are different from those of uniaxial compression. Moreover, this plane strain compression adopts lower heating rate and longer holding time, which can make the diffusion in the microstructure more sufficient and the alloy microstructure more uniform. This also reduces the deformation activation energy of the alloy. On the other hand, compared with the literature [36,37], the experiment in this paper is mainly carried out in the range of low strain rate, and the maximum strain rate is 0.05 s^−1^, which is far lower than 10 s^−1^ in the above literature. High strain rate will cause a large number of dislocation entanglement, resulting in the increase of deformation activation energy. In addition, compared with the as-cast plate selected in the literature [36,37], the rolled plate was selected and annealed at 1150 °C for 50 min. The obtained equiaxed microstructure has low deformation resistance. Due to different initial conditions, heat treatment process, and deformation parameters, the results of the deformation activation energy will change.

### 3.3. Thermal Processing Map

The hot workability of materials refers to the deformation ability of materials without damage in the process of plastic deformation. While the processing map reflects the processing advantages and disadvantages of materials under different processing conditions, which is related to the properties of materials [42]. During the hot working process, the work done by the external force (denoted by *P*) can be expressed by the plastic deformation (denoted by *G*) and the change of grain structure (denoted by J) [43]:

(15) $$P=\sigma \dot{\varepsilon }=G+J=\int_{0}^{\dot{\varepsilon }}\sigma d\dot{\varepsilon }+\int_{0}^{\sigma }\dot{\varepsilon }d\sigma$$

The relationship between *G* and *J* is described by Equation (16), where *m* is strain rate sensitivity index.

(16) $$m={\left(\frac{\partial J}{\partial G}\right)}_{\varepsilon ,T}=\frac{\partial P}{\partial G}\frac{\partial G}{\partial P}={\left[\frac{\partial ln\;\sigma }{\partial \dot{\varepsilon }}\right]}_{\varepsilon ,T}$$

When *G* equals *J*, *J* reaches the maximum *J_max_*. In order to describe the power dissipation characteristics of material microstructure evolution, we use efficiency of power dissipations η to represent it.

(17) $$\eta =\frac{J}{J_{max}}=\frac{P-G}{\sigma \cdot \frac{\dot{\varepsilon }}{2}}=2\left(1-\frac{\int_{0}^{\dot{\varepsilon }}\sigma d\dot{\varepsilon }}{\sigma \cdot \dot{\varepsilon }}\right)$$

When the strain is constant, the power dissipation diagram can be obtained from the contour map of temperature, strain rate and efficiency of power dissipations. However, according to the power dissipation diagram solely cannot reflect the actual processing situation. When the energy dissipation efficiency is high, instability may occur. Therefore, the power dissipation diagram has to be further improved.

Prasady et al. [44] considered that the dissipation function and strain rate satisfy the following inequality:

(18) $$\frac{dD}{d\dot{\varepsilon }}⩽\frac{D}{\dot{\varepsilon }}$$

When system is unstable, the criterion of instability of material is obtained as follows:

(19) $$\xi \left(\dot{\varepsilon }\right)=\frac{\partial lg\left(\frac{m}{m+1}\right)}{\partial lg\;\dot{\varepsilon }}+m⩽0$$

**Figure 8:**
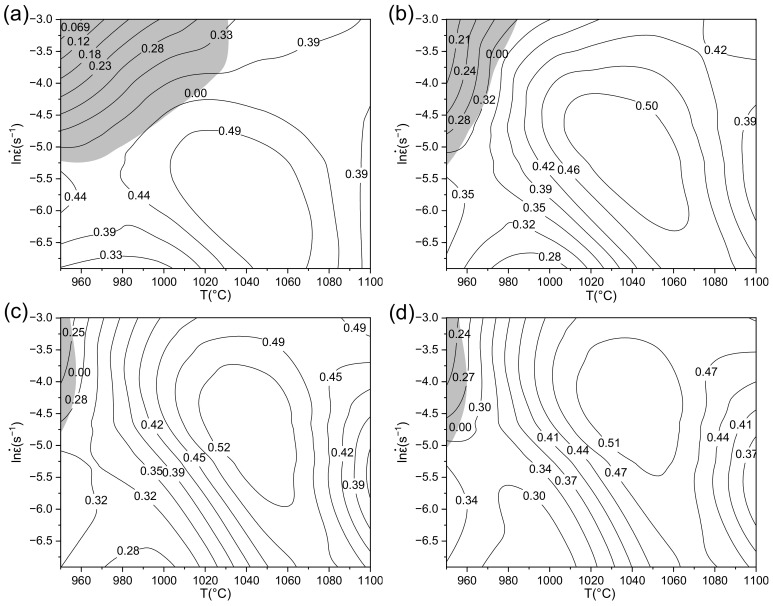
Thermal processing map of Inconel 625 under different effective strain. (**a**) ε = 0.2, (**b**) ε = 0.3, (**c**) ε = 0.5, (**d**) ε = 0.7.

Plot the unstable region on the power dissipation diagram, and the processing map can be obtained. The processing map under different effective strains is shown in Figure 8.

The shadow area in the figure is the unstable region, and the value of isoline is *η*. The region corresponding to the highest energy dissipation rate is the optimal deformation region. It can be seen that the unstable region is mainly concentrated in the range of low temperature and high strain rate, and with the increasing of temperature, the proportion of energy used for microstructure evolution raises. This is because the increase of temperature is conducive to the progress of DRX. As can be seen from Figure 8a, when the strain is low, the area of unstable region is large. At this time, the flow stress is near the peak value, and the recrystallization behavior has not been fully carried out. With the increasing of strain, the area of the unstable region gradually decreases, the optimal deformation zone moves to the high temperature and high strain rate zone, and the shape of hot working diagram tends to be stable. On the whole, the effect of strain on the change of hot working diagram is not great, so Inconel 625 can be regarded as a strain insensitive material. When the true strain is 0.7, the flow curve is basically stable. At this time, the optimum deformation condition is found to be 1020–1060 °C/0.005–0.03 s^−1^, and the maximum energy conversion can reach 0.51. The deformation temperature of 950–960 °C with the strain rate of 0.007–0.05 s^−1^ are determined as the unstable region.

**Figure 9:**
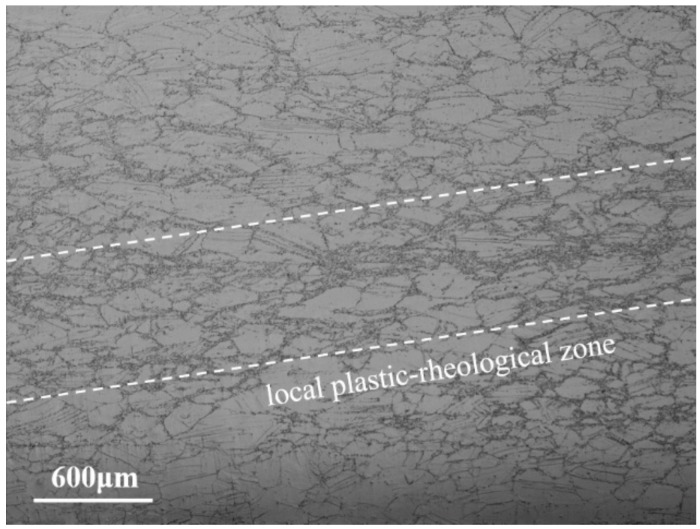
Microstructures of Inconel 625 under in the unstable region.

The specimens in the unstable region at the conditions of 950 °C/0.01 s^−1^ was selected for optical observation under metallographic microscope, and the results are shown in Figure 9. It can be seen that a banded recrystallization dense area appears in the sample. This is because under low temperature and high strain rate, the heat generated by local plastic deformation is too fast to be transmitted to the low temperature area, resulting in an instantaneous sharp rise in local temperature. The deformation resistance of this area decreases, and local large deformation occurs. All this promotes local DRX process, leading to the generation of local plastic-rheological zones. The above phenomena illustrate that the deformation of the alloy is very uneven during hot pressing in the unstable region, which indicates the characteristics of local instability. If the instability is further increased, adiabatic shear bands and microcracks may develop and cause sample failure. This phenomenon further verifies the guidance of the processing map. In the actual machining process, the unstable region should be avoided.

### 3.4. Microstructure Evolution of Inconel 625 during Thermal Deformation

**Figure 10:**
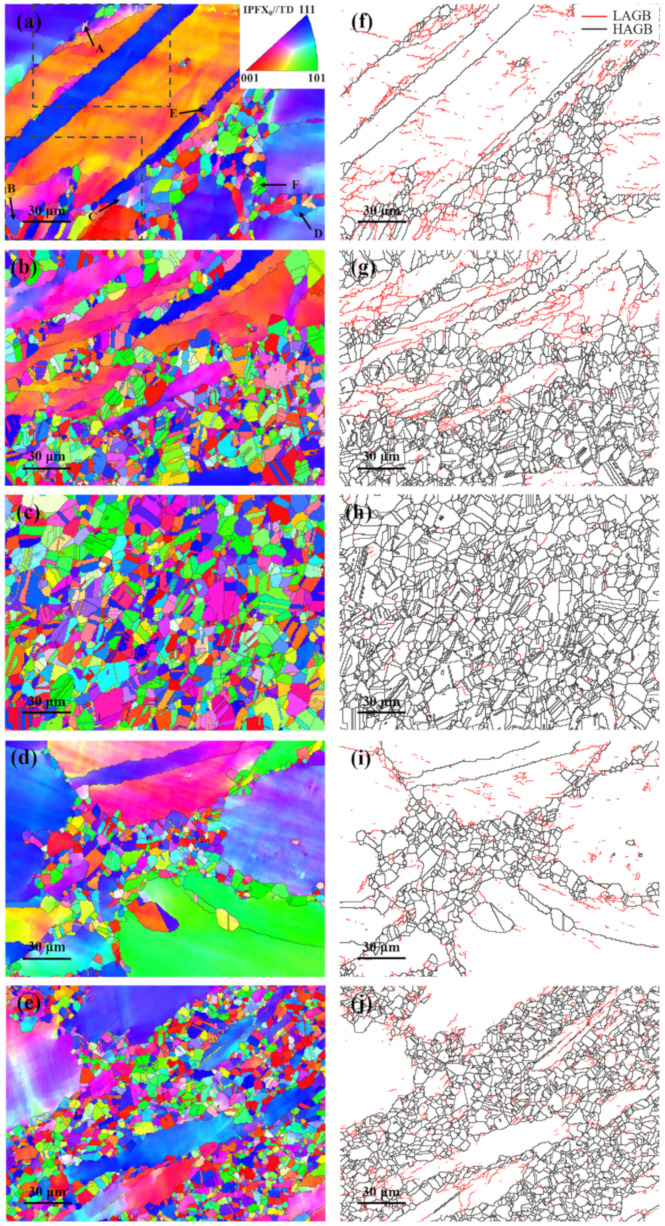
IPF maps and grain misorientations distribution of Inconel 625 under different hot-deformation conditions: (**a**,**f**) 1000 °C, 0.05 s^−1^, (**b**,**g**) 1000 °C, 0.01 s^−1^, (**c**,**h**) 1000 °C, 0.005 s^−1^, (**d**,**i**) 950 °C, 0.01 s^−1^, (**e**,**j**) 1050 °C, 0.01 s^−1^.

**Figure 11:**
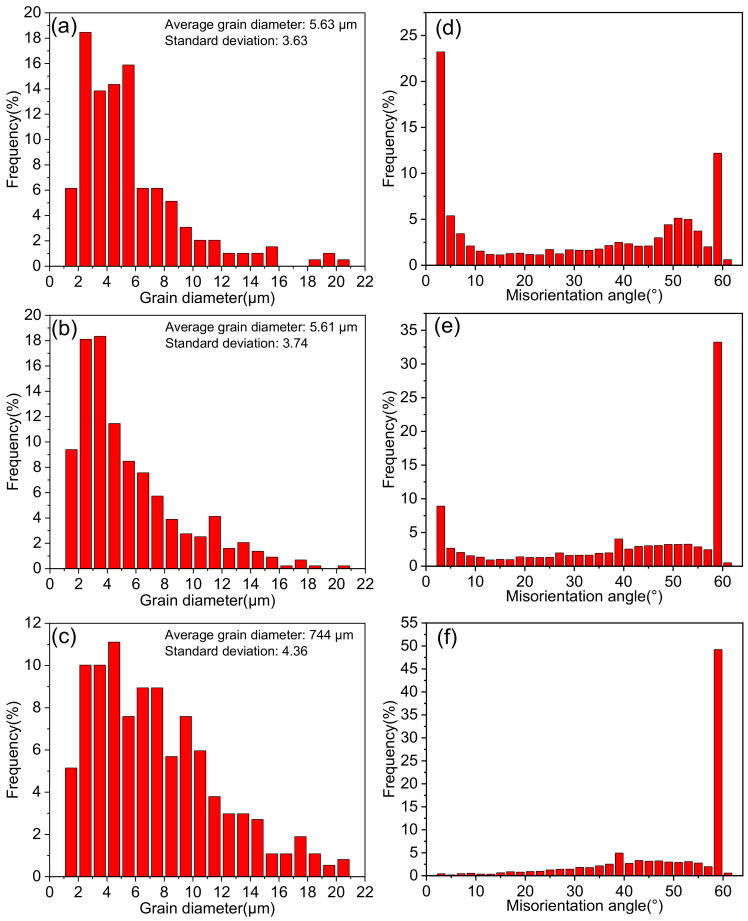
Grain sizes and misorientation angles under different strain rates: (**a**,**e**) 1000 °C, 0.05 s^−1^, (**b**,**d**) 1000 °C, 0.01 s^−1^, (**c**,**f**) 1000 °C, 0.005 s^−1^.

**Figure 12:**
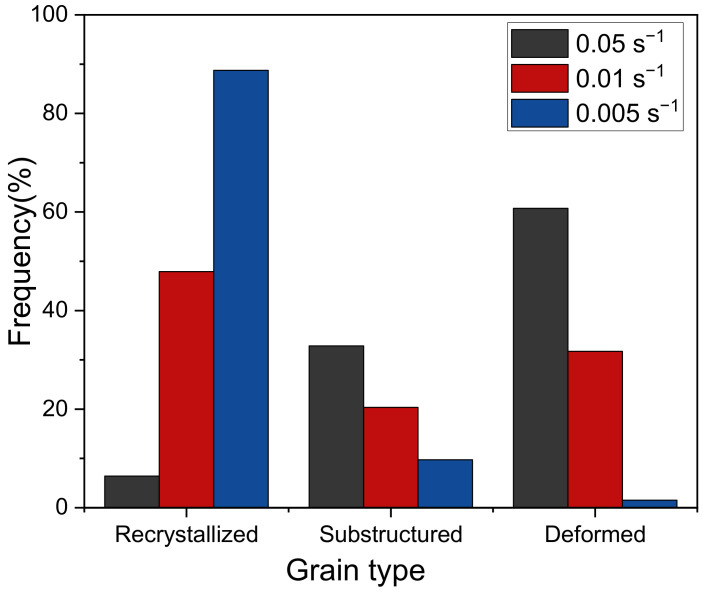
The volume fraction of different grains.

The Inconel 625 samples after the plane strain compression test were characterized by EBSD, and the results are shown in Figure 10. The misorientation angle of 2–15° is defined as a low angle grain boundary (LAGBs), and the misorientation angle greater than 15° is a high angle grain boundary (HAGBs). Figure 11 shows the quantitative statistics of grain sizes and misorientation angles at different strain rates. The noise below 1.5 μm and grains connected to the picture boundary are excluded through the subset function. The twin boundary is not included in the statistics, and the grain size is expressed by the equivalent diameter. In addition, Figure 12 shows the volume fraction of different grains.

From the Figure 10, Figure 11a and Figure 12 we can see under the temperature of 1000 °C and the strain rate of 0.05 s^−1^, the recrystallized grain is fine, and the coarse original grains are dominant. As shown in Figure 10, Figure 11b and Figure 12, with decreasing strain rate, the original coarse grains decrease gradually, and the grains with size of 0–5 μm increase rapidly. The fine recrystallized grains form uniformly, and twins appear in the grains. And Figure 10c shows when the strain rate further decreases, the recrystallized grains have enough time to grow. The original coarse grains almost disappeared, and the size of twin and recrystallized grains increases, as shown in Figure 11c and Figure 12. Therefore, whether the strain rate is too high or too low, it is unbeneficial for Inconel 625 to obtain fine grain structure during plane strain compression, which is unfavorable for the macro properties of the alloy. In the actual production, selection of the appropriate strain rate should be considered. The effect of deformation temperature on recrystallization structure is similar to that of strain rate. From Figure 10b,d,e, it can be found that the recrystallized grains of Inconel 625 are fine at low temperature. When the temperature rises, both the proportion and the size of recrystallized grains increase. This is consistent with the conclusion obtained from the stress–strain curve. As a result, the increasing temperature can promote the DRX behavior of the alloy.

In addition, it can be seen from Figure 10a that when the strain rate is low, some grain boundaries bend into a stepped shape due to stress concentration. These stepped grain boundaries hinder the movement of dislocations, resulting in the increasing of dislocation density. The nucleated DRX grains attached to these grain boundaries for nucleation preferentially. During the hot-pressing process, fine recrystallized grains surround the initial coarse grains and form chain structure. This is a typical sign of discontinuous dynamic recrystallization (DDRX). In the process of DDRX, there is a big difference between the initial grain size and the steady-state recrystallization grain size. The former is consumed by the latter in the form of alternate nucleation and grain growth. Through the analysis in Figure 11d–f, it can be seen that the distribution of the misorientation angle of Inconel 625 has bimodal characteristics. When the angle is less than 5° or about 60°, the frequency is the highest. In addition, with the decreasing strain rate, the low angle grain boundary decreases and the high angle grain boundary (mainly 60°) increases. This is related to the formation of annealing twins during thermal deformation. It is generally believed that the formation of annealing twins can reduce the boundary energy of growing grains and increase the mobility of grain boundaries [45]. Therefore, annealing twins can promote the dynamic recrystallization process.

**Figure 13:**
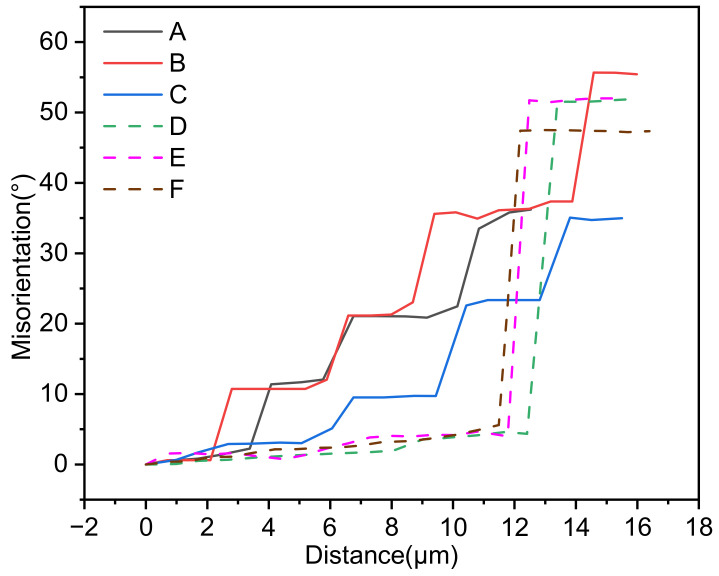
Cumulative misorientation along the arrows of Figure 10a.

**Figure 14:**
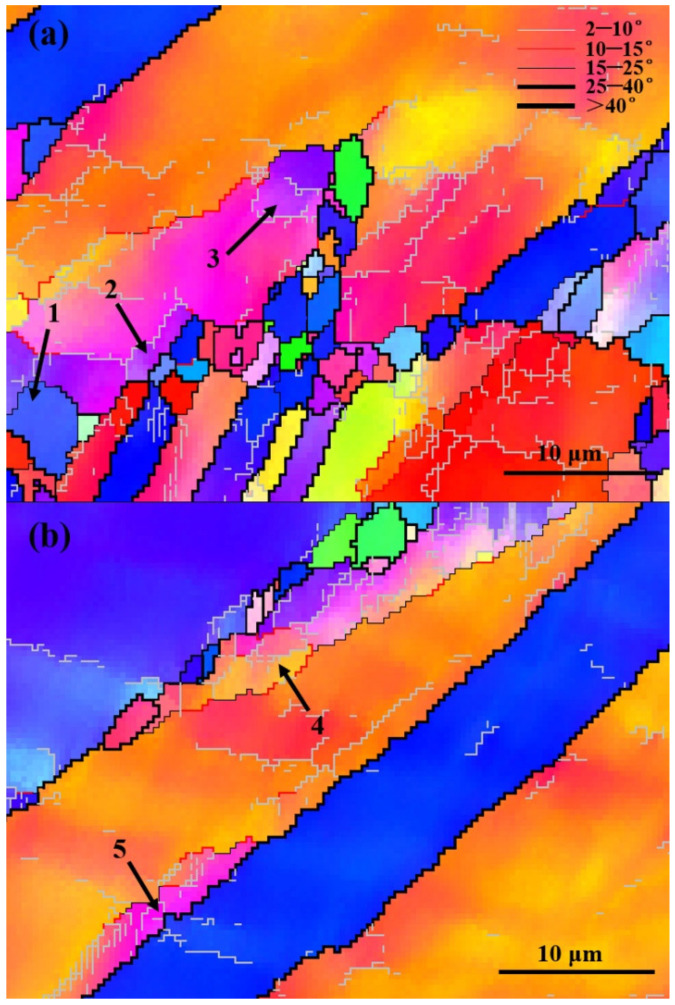
Partial enlarged view of the dotted line in Figure 10: (**a**) the lower part, (**b**) the upper part.

In order to explore the recrystallization mechanism of the Inconel 625 alloy at low strain rate, we take the cumulative orientation difference along arrows A–E in Figure 10a, and the results are shown in Figure 13. In addition, the partial enlarged view of the dotted line is shown in Figure 14.

**Figure 15:**
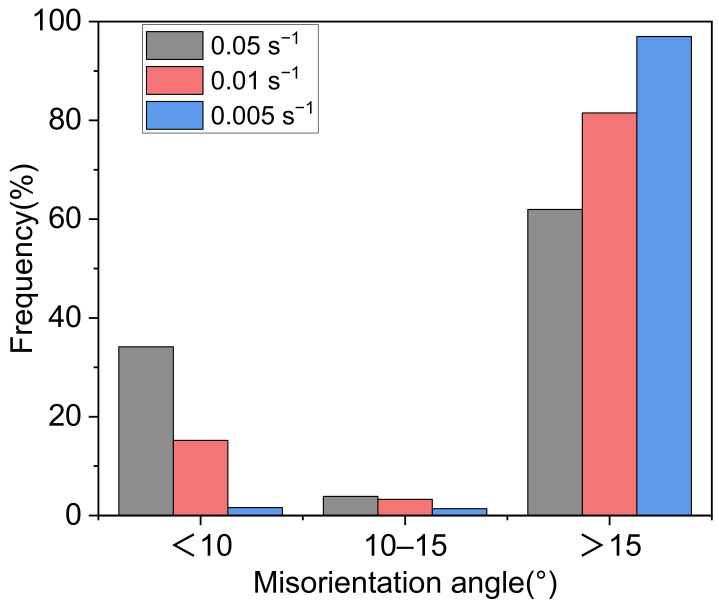
The changes in the fractions of different misorientation angles scopes at 1000 °C.

It can be found from Figure 13 that the cumulative misorientation at A–C increases in a step shape, indicating that the dynamic recrystallization grain here is formed by the evolution from a small angle grain boundary to a large angle grain boundary. And the grain boundary distribution in Figure 14 also proves this phenomenon, in which grains 1–5 are typical CDRX grains. On the other hand, the cumulative misorientation at D-F rises sharply when crossing the grain boundary, while in other regions it is flat; and the cumulative misorientation is much greater than the discrimination value of the large angle grain boundary by 15°. This phenomenon shows that the recrystallized grains here are formed by nucleation and growth through grain boundary bow, that is, discontinuous dynamic recrystallization, which shows that DDRX occurs. From the statistics of microstructure, we find that the number of recrystallized grains produced by CDRX is very small. To further illustrate this phenomenon, the changes in the fractions of different misorientation angle scopes under different deformation conditions are shown in Figure 15.

As can be seen from Figure 15, with the decreasing of strain rate, the LAGBs decrease gradually and remain in a small proportion. Under the above conditions, the proportion of LAGBs with an misorientation of 10–15° is always very low, and the proportion does not change significantly. It is well-known that the CDRX mechanism usually leads to an increase of 10–15° misorientation rate [46]. Therefore, under plane strain compression at high temperature and low strain rate, the DRX mechanism of the Inconel 625 alloy is DDRX based on grain boundary bulging and HAGBs migration. Meanwhile, the CDRX with sub-crystal merging and rotation is not dominant and plays an auxiliary role.

## 4. Summary and Conclusions

In this paper, a plane strain compression experiment on an Inconel 625 sheet under different thermal deformation conditions was carried out. By analyzing the stress–strain curves and microscopic characterization, we came to the following conclusions:

(1) Recrystallization occurs during hot pressing, and with the increasing of deformation temperature and the decreasing of strain rate, the recrystallization softening is more significant, so the stress level decreases. When the strain rate is higher, the curve is smooth. While the strain rate is low, the stress–strain curve shows high-frequency periodic oscillation.
(2) Calculating the related parameters, we get the constitutive equation of plane compression strain of an Inconel 625 sheet as $Z=\dot{\varepsilon }exp\left(2.58\times 10^{5}/RT\right)=1.06\times 10^{8}{\left[sinh\left(0.0037\sigma \right)\right]}^{3.21}$, and the average error is 5.68%.
(3) The processing map of the Inconel 625 sheet under different strains was drawn. It was found that the Inconel 625 is a strain-insensitive material. The unstable region is: deformation temperatures at 950–960 °C, and strain rates at 0.007–0.05 s^−1^. The best deformation region is: deformation temperatures at 1020–1060 °C, and strain rates at 0.005–0.03 s^−1^. Under the condition of instability, obvious local plastic-rheological zones can be observed, in which the fine DRX grains are densely distributed.
(4) Through EBSD characterization, it was found that increasing temperature and decreasing strain rate will promote the recrystallization behavior, and both the proportion and the size of recrystallized grains increase. Under the condition of high temperature and low strain rate, DDRX is the main deformation mechanism, while CDRX plays an auxiliary role.

## Figures and Tables

**Table 1:** Chemical composition of the Inconel 625 used in this work (wt.%).

Ni	Cr	Mo	Nb	Fe	Si	Al	Ti	Mn	C	S
61.00	21.50	9.00	3.60	2.00	0.20	0.20	0.20	0.20	0.05	0.001

## Data Availability

All data are available from the corresponding author on reasonable request.
